# Homology-independent targeted insertion (HITI) enables guided CAR knock-in and efficient clinical scale CAR-T cell manufacturing

**DOI:** 10.1186/s12943-023-01799-7

**Published:** 2023-06-26

**Authors:** Hyatt Balke-Want, Vimal Keerthi, Nikolaos Gkitsas, Andrew G. Mancini, Gavin L. Kurgan, Carley Fowler, Peng Xu, Xikun Liu, Kyle Asano, Sunny Patel, Christopher J. Fisher, Annie K. Brown, Ramya H. Tunuguntla, Shabnum Patel, Elena Sotillo, Crystal L. Mackall, Steven A. Feldman

**Affiliations:** 1 Stanford Center for Cancer Cell Therapy, Stanford Cancer Institute, Stanford University, Stanford, CA USA; 2 MaxCyte, Inc, Rockville, MD USA; 3 Integrated DNA Technologies, Inc, Coralville, IA 52241 USA

**Keywords:** CRISPR/Cas9, Homology-independent targeted insertion (HITI), Targeted insertion, Non-viral CAR-T cell, GMP, Genomic safety

## Abstract

**Background:**

Chimeric Antigen Receptor (CAR) T cells are now standard of care (SOC) for some patients with B cell and plasma cell malignancies and could disrupt the therapeutic landscape of solid tumors. However, access to CAR-T cells is not adequate to meet clinical needs, in part due to high cost and long lead times for manufacturing clinical grade virus. Non-viral site directed CAR integration can be accomplished using CRISPR/Cas9 and double-stranded DNA (dsDNA) or single-stranded DNA (ssDNA) via homology-directed repair (HDR), however yields with this approach have been limiting for clinical application (dsDNA) or access to large yields sufficient to meet the manufacturing demands outside early phase clinical trials is limited (ssDNA).

**Methods:**

We applied homology-independent targeted insertion (HITI) or HDR using CRISPR/Cas9 and nanoplasmid DNA to insert an anti-GD2 CAR into the *T cell receptor alpha constant* (TRAC) locus and compared both targeted insertion strategies in our system. Next, we optimized post-HITI CRISPR EnrichMENT (CEMENT) to seamlessly integrate it into a 14-day process and compared our knock-in with viral transduced anti-GD2 CAR-T cells. Finally, we explored the off-target genomic toxicity of our genomic engineering approach.

**Results:**

Here, we show that site directed CAR integration utilizing nanoplasmid DNA delivered via HITI provides high cell yields and highly functional cells. CEMENT enriched CAR T cells to approximately 80% purity, resulting in therapeutically relevant dose ranges of 5.5 × 10^8^–3.6 × 10^9^ CAR + T cells. CRISPR knock-in CAR-T cells were functionally comparable with viral transduced anti-GD2 CAR-T cells and did not show any evidence of off-target genomic toxicity.

**Conclusions:**

Our work provides a novel platform to perform guided CAR insertion into primary human T-cells using nanoplasmid DNA and holds the potential to increase access to CAR-T cell therapies.

**Supplementary Information:**

The online version contains supplementary material available at 10.1186/s12943-023-01799-7.

## Background

CAR-T cells are a novel drug class with impressive efficacy in refractory B-cell and plasma cell malignancies [1–3]. This success is fueling efforts to extend their efficacy to earlier lines of therapy [4] and for treatment of solid tumors [5–9]. All current commercially available CAR-T cells use viral vector based transgene delivery [10]. Access to these therapies is inadequate to meet clinical need, in part due to high costs and supply chain limitations related to the manufacturing and qualifying GMP grade vectors. Innovation in early stage trials is also limited by long lead times and high costs for production of viral vectors [11]. Non-viral gene delivery [12–17] could diminish the cost and complexity of cell manufacturing for CAR T cell therapies. CRISPR/Cas9 with adeno-associated virus 6 (AAV6) can deliver site-directed modifications of the genome via HDR, and this approach may reduce the risk of insertional mutagenesis compared to random insertions delivered via retroviruses and simultaneously knockout a gene of interest [18, 19]. In search of a viral free CAR-T cell manufacturing platform, investigators electroporated CRISPR/Cas9 Ribonucleoprotein (RNP) with linearized dsDNA or plasmid DNA for targeted transgene knock-in into T cells, but this has been associated with low efficiency and yield due to impaired T cell viability and expansion post editing, which has shown to impact clinical scale manufacturing [20–22]. More recently, truncated Cas9 target sequences (tCTS) added to ssDNA have enabled efficient knock-in of an anti-BCMA CAR without impairing cell yields [23]. However, access to ssDNA for large clinical trials and manufacturing post approval is limited. Additional fully non-viral CRISPR knock-in platforms are needed to ensure adequate access to meet the increasing demand for engineered T cells for therapeutic use.

HDR relies on cell division to provide a sister-chromatid chromosome copy created during S phase, which serves as the template for gene correction after a dsDNA break of the respective locus [24]. Because HDR is not the predominant DNA repair pathway utilized following dsDNA breaks, non-homologous end joining (NHEJ) mediated HITI has more recently been explored for large transgene insertions in both dividing and resting cells [25]. NHEJ is the primary DNA repair pathway utilized following dsDNA breaks and acts independent of the cell cycle state. DNA ends are ligated by Ligase IV after Ku proteins recruit nucleases to trim and polymerases to fill in gaps respectively [26]. Others have shown that HITI resulted in more efficient targeted knock-in when compared to HDR in adherent cell lines and embryonic stem cells, and the increased efficiency was more pronounced when using transgenes of large size (> 5 kb) [27, 28]. In vivo gene correction via HITI has been applied pre-clinically for treatment of retinitis pigmentosa, Mucopolysaccharidosis type VI and Adrenoleukodystrophy using either AAV6 or AAV9 [25, 29, 30]. Due to its cell cycle independent integration, HITI could expand the CRISPR knock-in toolbox for somatic cell and gene therapy [31], but it has not been explored for CRISPR knock-in into T cells.

Here, we tested HITI mediated site directed integration of a therapeutically relevant GD2-CAR transgene into the *T cell receptor alpha constant* (*TRAC*) locus using nanoplasmid DNA and CRISPR/Cas9 in primary human T cells. Compared to HDR mediated knock-in, HITI yielded at least 2-fold more GD2-CAR-T cells. When combined with CEMENT, HITI GD2-knock-in CAR-T cells were enriched 2.3-8 fold using dihydrofolate reductase^L22F/F31S^ (DHFR-FS), which confers resistance to the FDA approved drug methotrexate (MTX). Using a starting population of 5 × 10^8^ T cells and a 14-day process, HITI/CEMENT generated GD2-CAR-T cell numbers across 3 independent donors ranging from 5.5 × 10^8^—3.6 × 10^9^, sufficient to meet doses administered in all current commercial CAR products. HITI/CEMENT GD2 knock-in CAR-T cells showed an acceptable safety profile as assessed using ddPCR based copy number analysis, unbiased evaluation of off-target sites [32] and genome-wide insertion site analysis [33]. Furthermore, HITI/CEMENT GD2 knock-in CAR-T cells were functionally equivalent to viral transduced GD2-CAR-T cells and mediated tumor control of an in vivo model of metastatic neuroblastoma. This work has the potential for immediate clinical translation and could yield significant efficiencies for manufacturing of autologous engineered immune cell products.

## Methods

### Isolation and culture of primary human T cells

Fresh Leukopaks were obtained from STEMCELL Technologies and processed for negative selection using the EasySep Human T Cell Isolation Kit. Cells were activated using Dynabeads Human T-Activator CD3/CD28 (Thermo Fisher) at a 1:1 ratio and cultivated in TexMACS media supplemented with human IL-7 at 12.5 ng/ml and IL-15 at 12.5 ng/ml (all Miltenyi Biotec) as well as 3% human male AB Serum (Access Cell Culture). The culture volume was expanded over time to maintain cells at a concentration of ˜1.5 × 10^6^/ml using G-Rex 24-well and 6-well plates or G-Rex 100 M according to manufacturer’s instructions (Wilson-Wolf). Small molecule inhibitors AZD0156 (Selleck Chemicals) and Methotrexate (Sigma Aldrich) were resuspended in DMSO and added to the cell culture media as indicated.

### Nanoplasmid design and production

Nanoplasmid DNA was optimized for gene therapeutic application and consisted of two components: R6K origin of replication and an anti-Levansucrase antisense RNA to allow for an antibiotic-free selection. This ˜430 bp backbone prevents transgene silencing after genomic insertion [34–36]. Knock-In templates were synthesized at Genscript and flanking cut sites for NheI and KpnI were used to clone synthetic genes into nanoplasmid backbones. To introduce RNP cut sites within the Nanoplasmid DNA, the genomic target of the respective RNP was included into the above mentioned synthetic gene. Nanoplasmid DNA was manufactured at Nature Technology and resuspended at a concentration of 3 mg/ml in H2O. Nanoplasmid sequences can be found in Supplementary Table 1.

### sgRNA design

All gRNA sequences used in this study have been previously published [15, 16, 37, 38] and target: *TRAC* 5’- G**G**GAATCAAAATCGGTGAAT -3’, instead of 5’- G**A**GAATCAAAATCGGTGAAT -3’ and *B2M* 5’- CGCGAGCACAGCTAAGGCCA -3’, 5’– GAGTAGCGCGAGCACAGCTA -3’, 5’- GGCCGAGATGTCTCGCTCCG -3’.

### Electroporation

On day 2, unless otherwise indicated, Dynabeads were magnetically removed and cells were counted prior to electroporation on the Maxcyte GTx. For electroporation cells were washed once in Electroporation Buffer (Maxcyte) and then resuspended at 2 × 10^8^/ml for small scale experiments or in 2.4 ml for large-scale electroporation. Wildtype Cas9 (61 µM, IDT) and sgRNA (125 µM, IDT) were mixed vol 1:1 resulting in a molar ratio of 2:1 and incubated for 10 min. at room temperature. Hereafter indicated amounts of nanoplasmid DNA (3 mg/ml) were added to the RNP and incubated for at least 10 min. to allow the RNP to cut the nanoplasmid DNA. For small-scale experiments 5 × 10^6^ T cells were resuspended in 25 µl and 1.25 µl of RNP and respective amounts of nanoplasmid DNA were added. Cells were then transferred into OC-25 × 3 processing assemblies and electroporated using the Expanded T cell 4 protocol for activated T cells or the Resting T cell 14–3 protocol for electroporation of non-activated T cells, which were stimulated after electroporation with Dynabeads at a 1:1 ratio. Small scale experiments for GPC2 CAR knock-in were conducted using the OC100 × 2 processing assembly with a final reaction volume of 100 µl. For large scale experiments the GMP compatible CL1.1 assembly was used. Post electroporation cells were rested in electroporation buffer either in the processing assembly (OC-25 × 3, OC-100 × 2) or in G-Rex 6-well plates (CL1.1) for 30 min. before being transferred into final G-Rex vessels.

### Viral transduction

Our clinical grade GD2-CAR retroviral vector [5] was spinoculated on Retronectin (Takara) coated plates for 2 h at 3200 rpm on day 2. Hereafter, Dynabeads were removed from activated T cells and T cells were transduced at a MOI of 10 for 24 h.

### Post editing CEMENT

To enable enrichment of the desired CAR + knock-in population we optimized CEMENT based selection by comparing three different clinically relevant enrichment markers: Dihydrofolate Reductase^L22F/F31S^ (DHFR-FS), truncated Epidermal Growth Factor Receptor (tEGFR), and truncated Nerve Growth Factor Receptor (tNGFR) [9, 17, 39]. All three enrichment markers were successfully co-inserted along with the GD2-CAR resulting in total transgene sizes of ˜2.5-3 kb. CRISPR knock-in CAR-T cells were either enriched using the FDA approved drug MTX which was diluted from a 10 mM stock in media to result in a 50 nM final concentration. Successfully edited cells expressing the DHFR-FS protein are resistant to MTX while cells that did not harbor the CAR transgene stop proliferating. For comparison of column-based versus column-free surface marker selection GD2-CAR-tEGFR + cells were incubated with a primary antibody targeting tEGFR (Biolegend, clone: AY13) conjugated to Biotin. Hereafter successfully labeled T cells were either enriched using LS-columns and the QuadroMACS magnetic column separation system along with Streptavidin Microbeads (all Miltenyi Biotec) or using the EasySep Biotin Positive Selection kit for column-free separation (STEMCELL Technologies).

### Cell counts and viability

Cell counts and Viability were obtained using the Nexcelom Cellometer Auto 2000. Samples were mixed with AO/PI dye at a 1:1 volume ratio and then analyzed using the setting Immune Cells – Low RBC.

### Flow cytometry and intracellular staining

For flow cytometry 3–5 × 10^5^ cells were stained for 30 min. at 4C using commercially available antibodies as listed in Supplementary Table 2. For GD2-CAR and GPC2-CAR detection, an anti-14g2a idiotype or recombinant human Glypican-2 protein (R&D systems) were fluorescently labeled with the the DyLight 650 Microscale Antibody Labeling kit (Thermo Fisher). For intracellular staining CAR + T cells were co-cultured with respective tumor cell lines at a 1:4 ratio. Prior to co-cultivation the protein transport inhibitor Monensin (BD) and the anti-CD107a antibody (Biolegend) were added to T cell samples. 6 h after co-culture a surface staining was performed (30 min. at 4C), hereafter co-cultured cells were fixed for 50 min. at 4C and permeabilized to allow for intracellular staining over night at 4C using the Fixation/Permeabilization Solution Kit (BD Biosciences) as described previously [40]. Flow cytometry was performed on the CytoFLEX LX (Beckman Coulter).

### In vitro killing and ELISA assays

GD2-CAR-T cells from scale up experiments and GPC2-CAR-T cells from small scale experiments were harvested and co-cultured in 96-well plates at a 1:1, 1:5 and 1:10 ratio (normalized for CAR + T cells) with tumor cell lines Nalm6-GD2, CHLA255, SY5Y or SMS-SAN expressing GFP using 50,000 cells. Co-cultures were monitored for fluorescence signal using the Incucyte (Sartorius). For ELISA assays co-cultures were conducted at a 1:1 ratio and supernatant was collected 24 h later after centrifugation at 300 g for 10 min. ELISA MAX human IL-2 and IFN-g kits were purchased from Biolegend and co-culture supernatants were processed according to manufacturer’s instructions. Hereafter, plates were analyzed using the Thermo Scientific Varioskan Lux.

## Tumor cell line culture and In vivo studies

Nalm6-GD2, CHLA255, SY5Y and SMS-SAN tumor cell lines were cultivated in RPMI (Gibco) supplemented with 10% FBS (Sigma Aldrich) and 1% Penicillin/Streptomycin (Gibco). For in vivo studies SY5Y tumor cells were resuspended at 5 × 10^6^/ml in PBS and 200 µl (= 1 × 10^6^ tumor cells) were injected via tail vein injection into six- to ten-week-old female NOD-SCID γ^c−/−^ (NSG) mice. Mice were bred in house under Stanford University APLAC-approved protocols as described previously [41]. Seven days later 5 × 10^6^ GD2-CAR + T cells were injected via tail vein injection. Mice were euthanized when they manifested hunched posture, persistent scruffy co unless at, paralysis, impaired mobility, greater than 20% weight loss, if tumors interfered with normal bodily functions or if they exceeded limits designated in APLAC-approved protocols. Firefly luciferase expression was used to detect tumor activity over time. Bioluminescence images were taken after administration of 3 µg of D-Luciferin (15 µg/ml) by intraperitoneal injection. Images were either acquired on an IVIS imaging system 5 min. after injection using 30 s exposure times and medium binning or applying the auto-exposure setting. For data analysis all images were collected in a single file and analyzed on the Living Image software (Perkin Elmer).

### Genomic DNA extraction, IN&OUT PCR and Sanger sequencing

Genomic DNA was extracted using the PureLink Genomic DNA Mini Kit (Invitrogen). Primers flanking the insertion sites of HITI1c and binding outside the respective homology arm sequence of HDR2c as well as primers binding within the inserted sequences were designed using Primer3Plus (www.primer3plus.com). Primer sequences can be found in Supplementary Table 3. Phusion Hot Start Flex 2 × Mastermix (NEB) was used to amplify genomic regions of interest and DNA gel electrophoresis was conducted using the E-Gel Power Snap System (Thermo Scientific). Samples were shipped to ELIM BIOPHARM for Sanger sequencing. Sequences were aligned for analysis using snapgene.

### Post enrichment and in process cell counts from clinical products

In process cell counts were derived from post enrichment or day 2 samples of patients treated within NCT04196413, NCT04088890 and NCT03233854 [5, 40, 42]. Manufacturing was either conducted in-house at the Stanford Laboratory for Cell and Gene Medicine (LCGM) or at Miltenyi Biotec. All clinical studies and their amendments were approved by the Stanford Univerisity Institutional Review Board.

### ddPCR and copy number assessment

Extracted genomic DNA was digested using HindIII (NEB) and 10–66 ng of digested DNA were analyzed per sample. Samples were prepared using the Bio-Rad ddPCR Supermix for probes (no dUTP). Primer/Probe assays for Albumin (reference) and for detection of the TRAC-GD2-CAR insertion site (target) were designed using Primer3Plus (www.primer3plus.com) and purchased from IDT. Primer and Probe sequences can be found in Supplementary Table 3. For thermal cycling conditions we followed vendor recommendations (Bio-Rad, #1863024) and used an optimized annealing temperature at 58 °C. Droplets were generated using the QX200 manual droplet generator (Bio-Rad) and analyzed post PCR in the QX200 Droplet Reader (Bio-Rad). Samples were analyzed with QX Manager software using the automated threshold for digital cut-off. Copy numbers were normalized to the Albumin reference as well as GD2-CAR % and calculated as follows:

$$normalized\;CN=\frac{target\;copies\times2}{reference\;copies\times fraction\;of\;CAR\;expressing\;cells}$$

## Off-Target site prediction and quantification

Off-target sites were either described previously [15], or predicted using COSMID [43] and CCTop [44]. For COSMID the algorithm was set to allow for 3 mismatches and 1 Indel as described by Wiebking et al. [45] Additional off-target sites were predicted using CCTop which was adjusted to allow for a total of 4 mismatches, with 2 mismatches being concatenated. Primer pools were designed for multiplex PCR of off-target sites using the rhAmpSeq Design Tool (IDT) (https://www.idtdna.com/rhampseqdesigntool) and respective sites were amplified using the rhAmpSeq library kit (IDT) according to manufacturer’s instructions. Samples were pooled from all three independent donors to generate an equimolar ratio. The final sample was then sequenced at Novogene using MiSeq v2 chemistry (Illumina). Primer sequences of each off-target assay and indexing primers are listed in Supplementary Table 3 and a detailed list of off-target sites can be found in Supplementary Table 4. Editing events were quantified using CRISPAltRations utilizing default Cas9 parameterization, as described previously [15, 46]. Binary classification of off-target editing was performed using a thresholded Fishers Exact Test (*p* < 0.05) with limitations for site classification (% indels in treatment > 0.5%; % indels in control < 0.4%; > 5,000 reads per site) [15]. Products and tools supplied by IDT are for research use only and not intended for diagnostic or therapeutic purposes. Purchaser and/or user are solely responsible for all decisions regarding the use of these products and any associated regulatory or legal obligations.

## Insertion site analysis

For whole genome mapping of anti-GD2 CAR integration, edited cells were crosslinked and shipped to Cergentis B.V. for digestion, reverse crosslinking with ligation, PCR, subsequent sequencing and data analysis. NGS reads were aligned to the CAR sequence and the human genome (hg19 sequence) as described previously [33].

## Data analysis and software

Data analysis was performed using Microsoft Excel and GraphPad Prism. Statistical tests were conducted in GraphPad Prism and are indicated in the respective figure legend. Nanoplasmid and gRNA sequences were designed and confirmed in SnapGene (Dotmatics). We used FlowJo (FlowJo LLC) to analyze.fcs files derived from flow cytometry experiments. Living Image (PerkinElmer) was used for analysis of images derived from in vivo treatment of mice. Schematic illustrations were created in BioRender.

## Results

### HITI enables targeted CAR knock-in with enhanced CAR-positive cell yield

**Fig. 1:**
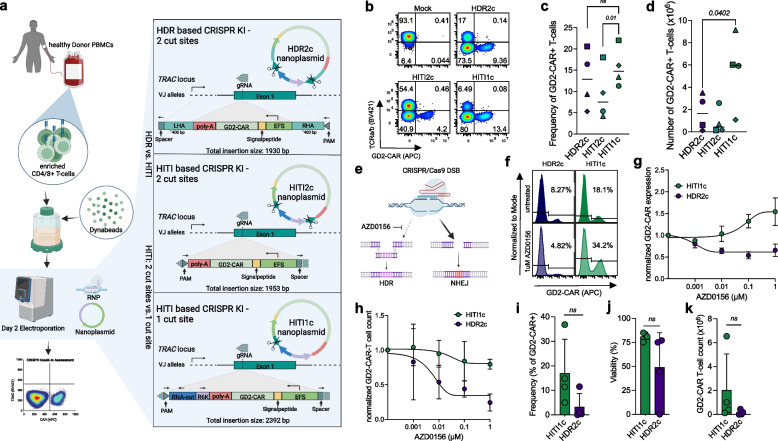
Comparison of Homology-Directed Recombination versus Homology-independent-targeted-insertion for targeted knock-in of a GD2-CAR into *TRAC*. **a** Schematic overview of workflow for experiments in b-d, and nanoplasmid designs for knock-in templates. **b**-**d** head-to-head comparison of constructs HDR2c, HITI2c and HITI1c. 5 × 10^6^ cells were electroporated per condition on day 2 post activation using respective constructs (0.75 µg of nanoplasmid per 1 × 10.^6^ cells) and analyzed via flow cytometry on day 10 (**b**, representative donor; **c**, pooled frequencies) and counted on the same day to assess GD2-CAR-T cell counts (**d**) (*n* = 4 independent donors). **e**–**h** HDR inhibitor induced modulation of GD2-CAR-T cell integration via HITI and HDR. **e** Schematic related to f–h. **f** Representative histograms of GD2-CAR expression after CRISPR knock-in with HDR2c or HITI1c templates either left untreated or treated with 1 µM of AZD0156 for 18 h post electroporation. **g** + **h** GD2-CAR expression (**g**) and GD2-CAR-T cell counts (**h**) normalized to untreated CRISPR knock-in samples after 18 h of treatment with indicated concentrations of AZD0156 (*n* = 3 independent donors). i-j, CRISPR knock-in of non-activated T cells using HITI1c and HDR2c for knock-in of the GD2-CAR. Indicated are knock-in frequencies (**i**), Viability (**j**) and GD2-CAR-T cell yield. Cells were counted and analyzed via flow cytometry on day 10 or 14 (*n* = 4 independent donors). *p* values were determined by paired two-tailed *t* tests. Error bars indicate standard deviation (SD)

We compared insertion efficiency of HITI vs. HDR using nanoplasmid DNA to deliver a GD2-CAR sequence into *TRAC*, which has been credentialled as a locus for targeted insertion (Fig. 1 a) [15, 18, 20, 47]. To maximize insertion efficiency and CAR expression in this system, we demonstrated that 0.75 µg nanoplasmid DNA per 1 × 10^6^ electroporated T cells maximized knock-in efficiency without impairing T cell viability (Supplementary Fig. 1 a and b), consistent with previous data using 0.5–1 µg linearized DNA per 1 × 10^6^ electroporated T cells [15].

Using an optimized nanoplasmid DNA concentration, we compared HDR vs. HITI mediated knock-in. For HITI we used two iterations with either one (HITI1c) or two cut sites (HITI2c). Consistent with previous reports, one cut site resulted in higher knock-in frequencies (mean frequencies HITI1c = 15.7% vs. HITI2c = 9.3%, *p* = 0.01) and yielded ~ sixfold more GD2-CAR T cells [25, 28]. On average, we observed ~ threefold higher GD2-CAR T cell yields and comparable insertion rates when using HITI1c vs. HDR2c (Fig. 1 b-d, Supplementary Fig. 7 a). We confirmed on target insertion of HDR2c and HITI1c constructs using IN&OUT PCR, which showed successful integration of the HITI1c nanoplasmid construct with 6 bp Indels at both junctions (Supplementary Fig. 2 a and b).

We further characterized HITI1c vs. HDR2c by interfering with HDR mechanisms. To promote HITI, we incubated T cells post electroporation with different concentrations of the ATM kinase inhibitor AZD0156, which has been described to inhibit HDR [48]. We observed decreased knock-in efficiency of HDR2c when AZD0156 was added, but higher insertion rates for HITI1c, validating the reliance on NHEJ in the HITI system. Nonetheless, we observed decreased GD2-CAR T cell yields in HITI1c samples after AZD0156 treatment compared to untreated controls (Fig. 1 f–h), and thus chose not include AZD0156 in the following experiments.

Given the cell cycle independent activity of the NHEJ repair pathway, we compared targeted genomic integration of the GD2-CAR using HITI1c and HDR2c in non-activated T cells and found higher insertion rates, viability and CAR + T cell yield after knock-in with HITI1c (Fig. 1 i-k). To validate that HITI mediated knock-in is versatile, we explored knock-in of our GD2-CAR into *B2M* (Supplementary Fig. 2 c-f), resulting in GD2-CAR T cell yields comparable to targeted insertion into *TRAC*.

## Optimization of CEMENT using clinical grade enrichment

To enhance purity of HITI GD2 knock-in CAR-T cells, we optimized CEMENT by comparing three enrichment systems compatible with clinical application, which incorporate human proteins to diminish the risk of immunogenicity, and utilize GMP grade reagents or clinically approved drugs. We tested immunomagnetic enrichment targeting tEGFR and tNGFR as previously described [9, 39], and drug based selection of cells expressing DHFR-FS, which confers resistance to the approved drug methotrexate (MTX) [49, 50]. To optimize conditions for surface marker-based selection we compared column-based versus column-free magnetic selection and optimized the timing of enrichment. We noted higher purities using a column-based magnetic selection (Supplementary Fig. 3 a and b) and higher viabilities and yields when enriching on day 9 of our culture (Supplementary Fig. 3 c-f).

**Fig. 2:**
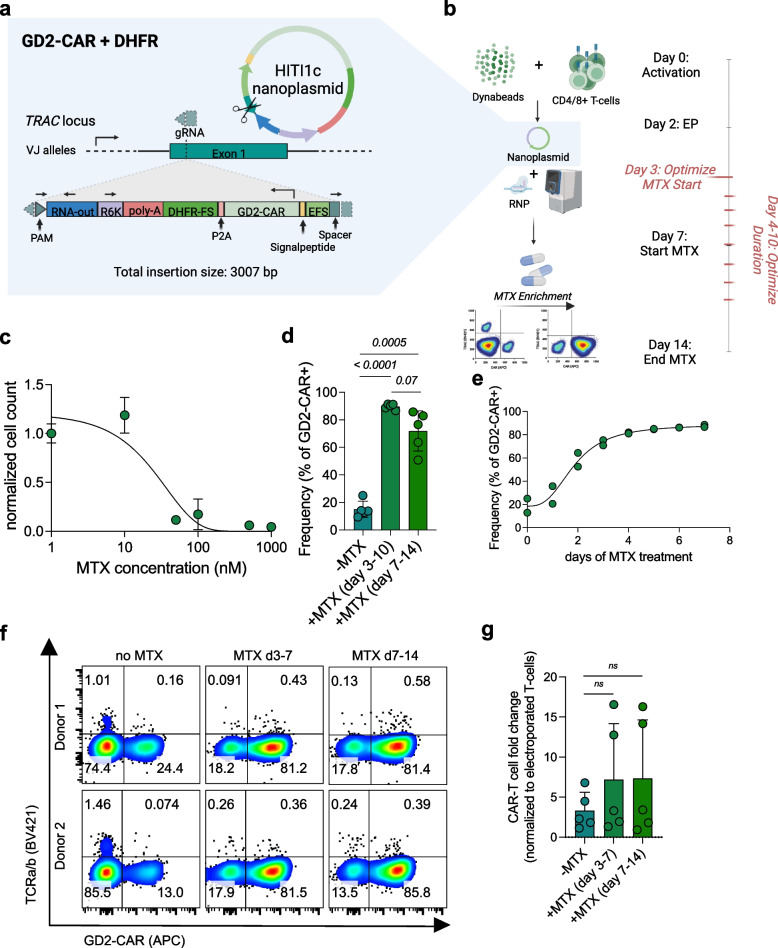
Optimization of Methotrexate (MTX) based selection of CRISPR knock-in GD2-CAR-DHFR-FS T cells. **a** GD2-CAR-DHFR-FS nanoplasmid design incorporating a gRNA cut site for linearization of the nanoplasmid and dsDNA break in *TRAC* with correct transgene insertion indicated. **b** Experimental layout for optimization of MTX enrichment. MTX treatment from day 7–14 has previously been reported to result in efficient enrichment in viral transduced CAR-DHFR-FS T cells and served as a reference. **c** Titration of MTX in primary human T cells with efficient killing starting at 50 nM MTX (*n* = 2 independent donors analyzed in technical duplicates). **d** Comparison of knock-in frequency determined via flow cytometry on day 14 in GD2-CAR-DHFR-FS T cells either non-enriched, enriched from day 3–10 or from day 7–14 (*n* = 5 independent donors). **e** MTX time course after CRISPR knock-in starting on day 3 for up to 7 days with plateaued enrichment after 4 days of treatment. All samples were assessed via flow cytometry on day 14 (*n* = 2 independent donors). **f** Quadrant plots indicating TCR-a/b and GD2-CAR surface expression for two representative out of five independent donors non-enriched, enriched from day 3–7 and from day 7–14. Flow cytometry was conducted on day 14. **g** GD2-CAR-T cell yield at day 14. Fold changes were calculated based on number of electroporated T cells on day 2 (*n* = 5 independent donors). Experiments in d and g were evaluated for statistical significance by paired, two-tailed *t* tests. Error bars indicate SD

We next optimized timing and duration of selection using MTX (Fig. 2 a and b), first by exposing primary human T cells post activation to varying concentrations of MTX and confirming that MTX exerts cytostatic effects in dividing T cells at 50 nM (Fig. 2 c). We added MTX to the test condition on day 3 or day 7 as reported previously [49] (Fig. 2 b) and observed improved enrichment of GD2-CAR-T cells when MTX was added on day 3 (range 86.6–91.4%) compared to day 7 (range 50.8–85.7%) (Fig. 2 d). We next shortened the duration of MTX exposure, and observed increased GD2-CAR frequencies with treatment durations of up to 4 days, resulting in ˜80% purity (range 76–81.5%), followed by a plateau for days 5–7 (Fig. 2 e). We next compared MTX based enrichment (optimized day 3–7 schedule) with surface marker based enrichment using tEGFR or tNGFR of GD2 knock-in CAR-T cells. The platforms were comparable in terms of purity and viability assessed 14 days after T cell activation. However, we observed ˜40-fold higher GD2-CAR-T cell recovery following DHFR-FS based enrichment (Supplementary Fig. 4 a-c). Together, our optimized CEMENT procedure using MTX enrichment of GD2-CAR-DHFR-FS knock-in cells comprises a 14-day process to manufacture feeder-cell-free, non-viral CRISPR knock-in CAR-T cells, and shortened MTX exposure compared to previously published schedules for enrichment of viral transduced and megaTAL/AAV6 modified T-cells [49, 51] (Fig. 2 f and g). Furthermore, we extended our work to generate GPC2 knock-in CAR-T cells using HITI/CEMENT resulting in knock-in efficiencies ranging from 5.5%—9.7% for non-enriched and from 41.4%—63.2% for enriched GPC2 knock-in CAR-T cells as determined on day 10 (Supplementary Fig. 5 a + b). GPC2 knock-in CAR-T cell counts showed a mean 1.54-fold increase over electroporated enriched T-cells by day 14 (Supplementary Fig. 5 c).

### HITI and CEMENT enable dose relevant manufacturing

**Fig. 3:**
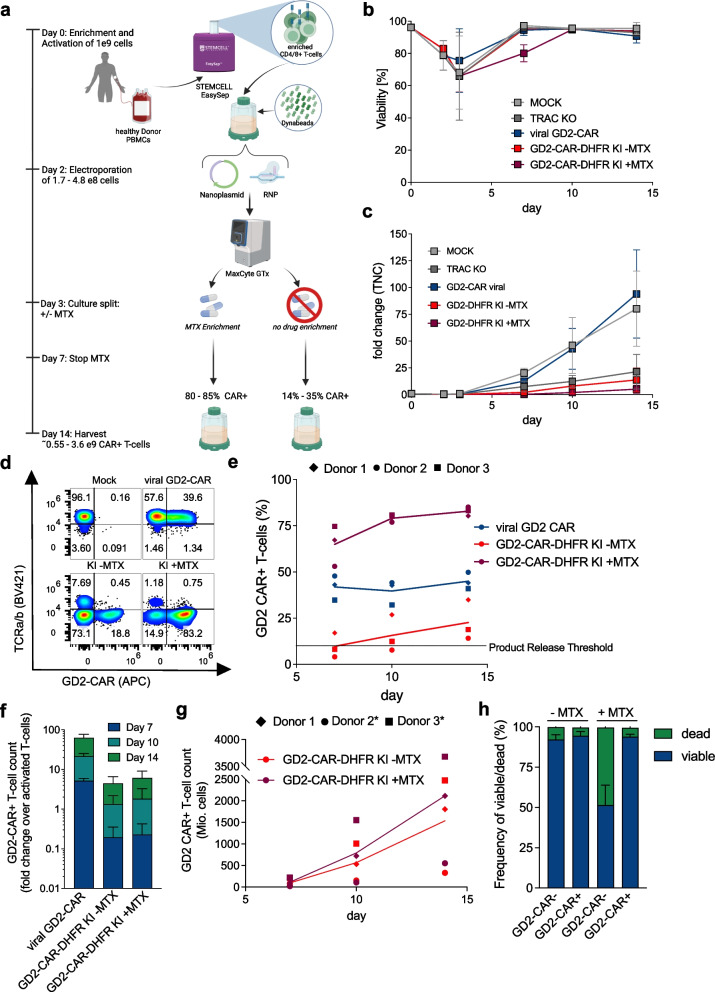
HITI based CRISPR knock-in CAR-T cell manufacturing at clinical scale. **a** Schematic workflow of leukapheresis processing to manufacture CRISPR KI CAR-T cells at clinical scale. Per Donor 1 × 10^9^ cells were activated and electroporated. Cultures were split up equally and either left untreated or treated with MTX for enrichment. **b** + **c** Viability (**b**) and fold change (**c**) of respective cultures over time. **d** Representative quadrant plots (day 14) showing GD2-CAR expression in *TRAC* positive cells for viral transduced CAR-T cells and *TRAC* negative cells for GD2 knock-in CAR-T cells. **e** GD2-CAR frequency over time across all three donors. **f** expansion of respective GD2-CAR-T cells for different time points normalized to the number of activated T cells. g, Total GD2-CAR-T cell counts for knock-in CAR-T cells (* = Donors with projected numbers after culture split on day 10). **h** Frequency of viable and dead cells in edited and non-edited T cells after MTX treatment assessed via flow cytometry on day 7. All experiments were conducted with *n* = 3 independent donors. Error bars indicate SD

Given the high frequencies and yield achieved using HITI mediated CAR knock-in and DHFR-FS based enrichment at laboratory scale, we tested our novel manufacturing platform at clinical scale. Based on the numbers of enriched T cells obtained from processed leukapheresis from diseased patients treated on ongoing clinical trials at our institutions (NCT04196413, NCT04088890, NCT03233854) [5, 40, 42] (Supplementary Fig. 6a), we activated 1 × 10^9^ enriched T cells using CD3/CD28 Dynabeads and cultivated T cells in G-Rex. On day 2, activated T cells were electroporated using Maxcyte’s GTx with the GMP compatible CL1.1 processing assembly to knock-in the GD2-CAR-DHFR-FS HITI1c construct into *TRAC.* T cell counts on day 2 prior to electroporation ranged from 1.7–4.8 × 10^8^ due to contraction and overlapped with historical day 2 cell counts obtained from patients treated on NCT04196413 which uses a similar activation process prior to retroviral vector based GD2-CAR delivery (Supplementary Fig. 6b + c) [5]. On day 3, HITI knock-in GD2-CAR-T cells were equally split and maintained in media containing 50 nM MTX or no drug. On day 7, media was replaced in the MTX enriched cultures and CAR-T cells were harvested on day 14 (Fig. 3 a). Samples obtained during manufacturing revealed that cell viabilities recovered to > 90% by day 7 in all conditions apart from MTX enriched cultures, where cell viability reached > 90% by day 10 (Fig. 3 b). Total fold change of T cells indicated expansion in edited cell samples (Fig. 3 c).

Flow cytometry of cells analyzed on days 7, 10 and 14 showed targeted insertion with HITI GD2-CAR-T cells and successful enrichment after MTX treatment (Fig. 3 d). On day 14, CAR frequencies ranged from 14–35% (non-enriched) and 80–85% (enriched) respectively (Fig. 3 e) with 3-4 fold expansion of HITI GD2-CAR-T cells compared to the number of initially activated T cells, on average resulting in a total of 2.1 × 10^9^ (range: 5.5 × 10^8^–3.6 × 10^9^) GD2-CAR-T cells in the enriched cultures (Fig. 3 f + g). These results demonstrate successful scale up of HITI mediated targeted transgene insertion and CEMENT. Last, we explored the effects of MTX on viability of edited vs. non-edited T cells using a reversed gating strategy (Supplementary Fig. 7 b) and observed that only non-edited cells were compromised in their viability at the end of MTX treatment (day 7) (Fig. 3 h), demonstrating that DHFR-FS successfully confers resistance against MTX. In addition, an extended culture using media either supplemented with or without cytokines (IL-7 + IL-15) did not show any cytokine independent outgrowth of DHFR-FS expressing cells independent of MTX exposure (Supplementary Fig. 6 d and e).

### Functional comparison of knock-in and transduced CAR-T cells

**Fig. 4:**
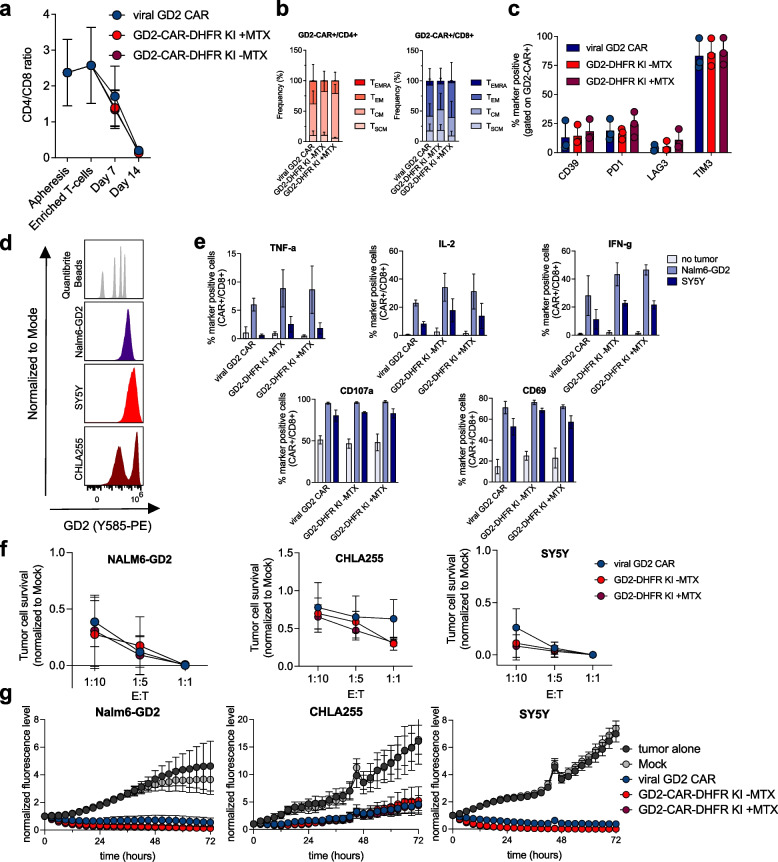
Knock-in GD2-CAR-T cells do not show phenotypic differences and are not functionally inferior when compared to viral GD2-CAR-T cells. **a** Changes of CD4/CD8 ratio after processing of leukopaks and over time. **b** + **c** Memory marker (**b**) and exhaustion marker (**c**) expression of viral vs. GD2 knock-in CAR-T cells as determined via flow cytometry on day 14 (pooled data from *n* = 3 independent donors). **d** GD2 antigen levels of co-cultured tumor cell lines. Representative histograms from *n* = 3 independent experiments. **e** Intracellular cytokine (TNF-a, IL-2, IFN-g) and activation marker (CD107a, CD69) expression after 6 h of co-culture with respective GD2 expressing tumor cell lines. Shown here is the marker positive cell frequency gated on CD8 + CAR + T cells (pooled data from *n* = 2 independent donors tested in technical triplicates). **f** Concentration dependent tumor cell killing after 48 h of co-culture with indicated E:T ratios (pooled data from *n* = 3 independent donors). **g** Tumor cell killing over time in GD2 antigen expressing tumor cell lines at a E:T ratio of 1:10 (pooled data from *n* = 2 independent donors tested in technical triplicates). Error bars indicate SD

**Fig. 5:**
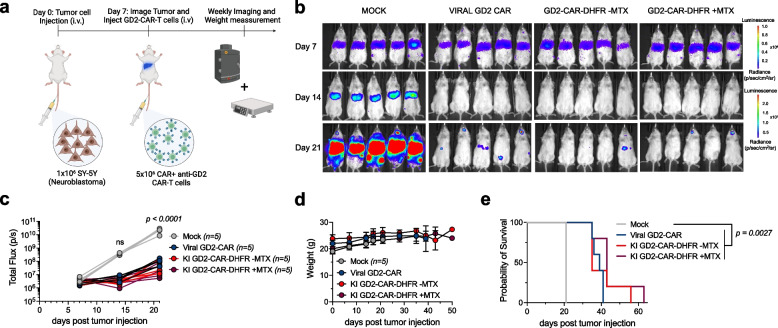
Knock-in GD2-CAR-T cells efficiently control growth of the SY5Y metastatic Neuroblastoma in vivo model. **a** Schematic of SY5Y tumor cell injection (1 × 10^6^ on day 0 via tail vein injection) in NSG mice. Confirmed tumor engraftment on day 7 via bioluminescent imaging and consecutive GD2-CAR-T cell treatment on day 7 using 5 × 10^6^ GD2-CAR-T cells applied via tail vein injection followed by weekly imaging of tumor bioluminescence. **b** Bioluminescent images of treated mice over time with color encoded radiance (p/sec/cm^2^/sr). **c** Total flux values (p/s) of all animals over time. Statistical significance was evaluated using two-way ANOVA multiple comparisons along with Dunnett’s test for indicated time points. **d** Weight of all treated animals over time without relevant changes over baseline. **f** Kaplan–Meier Survival analysis of treated animals. Statistical significance was evaluated using Mantel-Cox test. Error bars indicate SD

To understand how knock-in compare to viral transduced GD2-CAR-T cells, we analyzed CD4/8 ratio, memory phenotype and exhaustion profile and observed no significant differences (Fig. 4 a-c, Supplementary Fig. 7 c). We observed a clear bimodal distribution of CAR expression for the knock-in CAR-T cells with a slightly higher MFI and a significantly lower coefficient of variation compared to virally transduced GD2-CAR-T cells (Supplementary Fig. 8 a-c). To compare functionality of GD2-CAR-T cells generated by viral transduction vs HITI/CEMENT, we performed co-culture experiments using GD2 + tumor cell lines (Fig. 4 d, Supplementary Fig. 8 d), and observed similar activation marker expression, cytokine production and tumor cell killing at low E:T ratio (1:10) by viral and knock-in GD2-CAR-T cells (Fig. 4e-g, Supplementary Fig. 8 e–g). We next tested performance of HITI/CEMENT CAR-T cells in an in vivo model of metastatic neuroblastoma. We injected 1 × 10^6^ SY5Y tumor cells via tail vein injection and confirmed tumor lesions in liver and bone marrow on day 7. Hereafter, 5 × 10^6^ CAR + T cells were injected via tail vein injection. Tumor burden was tracked by weekly live imaging and weight measurements to assess toxicity (Fig. 5 a). While Mock T cell treated tumors did not show any disease control, all treatment arms showed comparable and significantly delayed tumor growth without evidence of toxicity (Fig. 5 b-e).

### Off-target genomic toxicity assessment in knock-in CAR-T cells

**Fig. 6:**
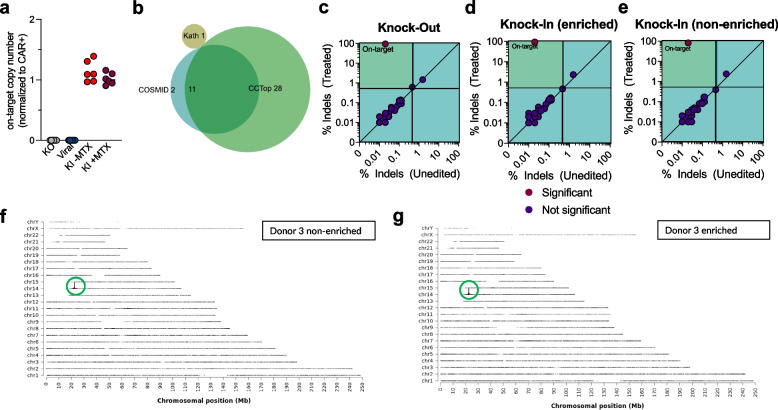
Genomic characterization of CRISPR knock-in CAR-T cells. **a** On-target copy number estimation using ddPCR. Genomic DNA from scale up experiments (*n* = 3 independent donors) was analyzed in technical duplicates using primers/probe to target Albumin (reference gene, copy number = 2) and primers/probe to target the left insertion site of the GD2-CAR into *TRAC*. Copy number values were normalized to the frequency of CAR + cells as determined via flow cytometry. **b** Source of predicted off-target sites. c-e, Quantification of indels in predicted off-target sites and *TRAC* using CRISPAltRations for samples obtained from Donor 3 of large-scale experiments. Editing was binarily classified using a thresholded Fishers Exact test (*p* < 0.05) with limitations (> 0.5% indels in treatment; < 0.4% indels in control; > 5,000 reads) for edited samples with (**c**) knock-out, (**d**) knock-in without enrichment and (**e**) knock-in after enrichment (red circle = significant; blue circle = not significant). Indel frequencies were plotted against non-electroporated Mock control samples to highlight pre-existing indels and noise. Quadrants display the limits of classification (bottom left – treatment % indels < 0.5; top right – control % indels > 0.4%; top left – all limitations met and classifiable; bottom right – no limitations met). The top left quadrant contains classifiable events that occur in edited samples and indicates only on-target editing in these samples. **f** + **g** Representative insertion site analysis for Donor 3 samples of non-enriched (**f**) and enriched (**g**) GD2 knock-in CAR-T cells using TLA. GD2 CAR sequences were inserted into the *TRAC* locus on chromosome 14 without evidence for off-target insertion

We assessed copy number levels of GD2-CAR utilizing ddPCR, which showed an average copy number for GD2 knock-in CAR-T cells of 1.08 (Fig. 6 a). To identify off-target cut sites introduced by the CRISPR/Cas9 RNP, we utilized a gRNA previously optimized for off-target cutting as assessed via GUIDE-Seq [15] and we also predicted additional off-target cut sites by applying the in silico prediction tools COSMID and CCTop [43, 44] (Fig. 6 b). All predicted cut sites are located outside exonic sequences (Supplementary Fig. 9 a) and were quantified using rhAmpSeq [32]. We conducted off-target evaluation in Mock, Knock-out, Knock-In -MTX and Knock-In + MTX samples of all three donors studied during our large-scale experiment. Quality control confirmed high quality sequencing data with a median coverage of 36,090 × and 99.1% of sequenced sample sites exceeding 5,000 × coverage (Supplementary Fig. 9 b-d). To classify off-target editing binarily, we applied a thresholded Fishers Exact test on paired treatment/control data with a limit of detection of 0.5% indels, as previously described [15] and compared edited samples against Mock control conditions for each respective donors. We identified significant and biologically relevant editing at the on-target site, i.e. *TRAC*, but no off-target editing was detected, indicating that neither CRISPR knock-in via HITI nor the MTX based enrichment procedure increased the risk of off-target editing (Fig. 6 c-e, Supplementary Fig. 9 e–g). To confirm that our approach results in site-directed knock-in, we performed targeted locus amplification (TLA) as described previously [33] using non-enriched and enriched samples from all three large scale runs. This unbiased genome-wide insertion site analysis has been widely accepted in the field [20, 52] and confirmed that our HITI/CEMENT approach inserts without off-target integration (Fig. 6 f + g, Supplementary Fig. 10).

## Discussion

This work provides a new approach for clinical scale manufacturing to deliver CAR knock-in to human T cells using a fully non-viral CRISPR/Cas9 based platform. Here, we show for the first time that plasmid DNA mediated HITI is feasible for targeted transgene insertion into primary human T cells and demonstrate that HITI combined with CEMENT results in clinically relevant CAR-T cell yields, providing an efficient and genotoxicity-free clinical scale manufacturing process (Fig. 1 d, Supplementary Figs. 2 b, 3 and 7). The process utilizes reagents available in sufficient quantities at reasonable cost to support both early proof-of-concept trials and potentially commercial manufacturing of CAR-T cell products. Our approach utilized nanoplasmid DNA as a delivery platform, which based upon our experience with research grade templates provided from vendors, is available at least 4-times quicker than dsDNA and is ˜20-fold cheaper. Furthermore, a recent report indicates nanoplasmid DNA to be more efficient in transgene delivery than dsDNA [47]. Nanoplasmid DNA can be manufactured in batches beyond 1 g, and only 125-360 µg of nanoplasmid DNA was utilized per product in this work (Supplementary Fig. 6 c), raising the prospect that one batch of nanoplasmid could provide sufficient template DNA to manufacture CRISPR knock-in CAR-T cells for a few thousand patients [35]. A clinical trial (NCT03970382) published by Foy et al. indicated the feasibility of utilizing nanoplasmid DNA as donor template within a clinical manufacturing context. The authors used nanoplasmid DNA to manufacture non-viral CRISPR knock-in neoantigen-specific TCR (neoTCR) T-cell products via HDR, which after optimizing their manufacturing process and incorporating a pre-commercial electroporation device, resulted in acceptable knock-in frequencies and yields of their CD8 + neoTCR T-cells [52].

Here, we used a HDR construct with two internal RNP cut sites as has been proposed recently [21, 31]. During our studies, two other groups however published data, successfully utilizing nanoplasmid DNA based HDR templates without internal RNP cut sites [47, 52]. When comparing optimized HITI vs. HDR in our system utilizing internal template cut sites and an external promoter for comparable transgene expression between HITI and HDR templates, we observed that insertion efficiencies were more consistent with HITI1c and independent of the cell cycle state based upon similar efficiencies with activated and non-activated T cells (Fig. 1 b, c, i). Further, yield of GD2-CAR-T cells was higher when using HITI1c versus HDR2c (Fig. 1 d and k). The basis for this finding remains unclear, but could be explained by the mechanism of transgene insertion. While for HITI the nanoplasmid DNA provided during electroporation is incorporated directly into the genome, for HDR the nanoplasmid DNA serves as a template for the endogenous HDR machinery and leaving remaining post-insertion exogenous HDR nanoplasmid DNA that could exert toxic effects on successfully edited T-cells and thereby reducing CAR-T cell yield (Supplementary Fig. 2 a and b). An alternative approach to improve CAR-T cell yields has recently been proposed [23] and relies on the combination of tCTS [53] and ssDNA donor templates, which are known to be less toxic than dsDNA templates [20]. By adding tCTS to the ends of ssDNA templates, RNP will bind to the ssDNA and via the nuclear localization sequence of Cas9 promote shuttling of the donor DNA into the nucleus helping to improve the historically low insertion efficiencies of ssDNA. However, tCTS have not been successful when integrated into dsDNA / nanoplasmid DNA templates by other groups [15, 47] and we anticipate that access to large yields of ssDNA will be limited.

Our data demonstrates that delivery of DHFR-FS protein provides a powerful tool for MTX based enrichment of edited T cells, generating GD2 CAR-T cell products with purities of at least 75%. It confirms that post editing DHFR-FS expression renders primary human T cells resistant to nanomolar doses of MTX as has been shown with megaTAL/AAV6 edited human CD4 + T-cells enriched for cells successfully edited at the CCR5 locus (Fig. 3 h) [51]. While our scale up work used healthy donors for manufacturing, the contraction seen on day 2 across all three donors used in our study aligned well with changes in cell numbers observed using viral-mediated CAR-T cell manufacturing on day 2 within NCT04196413 [5] (Supplementary Fig. 6 b). Of interest, one of the donors used in our study showed a massive contraction resulting in only 17% of activated T cells remaining prior to electroporation (Supplementary Fig. 6 c). Despite this, the HITI/CEMENT approach yielded a clinically relevant number of CAR-T cells for this donor (Fig. 3 g). Together, the data demonstrate that HITI/CEMENT CAR-T cells and virally generated GD2-CAR-T cells have similar antitumor activity in vivo (Figs. 4 and 5), without evidence for genotoxicity, and can be produced at clinical-scale (Fig. 6 and Supplementary Figs. 9+10).

## Conclusion

We have developed and optimized HITI/CEMENT, a new platform that allows precise genome engineering and enrichment of T cells. HITI/CEMENT delivered high yields of anti-GD2-CAR-T cells, sufficient for clinical application. We chose nanoplasmid DNA as our delivery platform due to its lower cost per product compared to linearized DNA templates. Our approach provides new avenues to explore HITI within the context of activator-free ex vivo manufacturing of CAR-T cells or in situ generation of CAR-T cells [54, 55].

## Supplementary Information

**Additional file 1:** **Supplementary Fig. 1. **Concentration optimization of CRISPR knock-in nanoplasmid constructs. a, Cell viability after electroporation with indicated amounts of nanoplasmid per 1x10^6^ cells. b, GD2-CAR expression after electroporation with indicated amount of nanoplasmid (*n* = 2 independent donors). **Supplementary Fig. 2.** CRISPR knock-in using HITI1c integrates electroporated nanoplasmid DNA and can be applied in a versatile manner. a, IN&OUT PCR of genomic DNA extracted from Mock, HITI1c and HDR2c samples using primers targeting the endogenous *TRAC* sequence outside homology arms of HDR2c and sequences within knock-in templates. b, Mapped Sanger Sequencing results from HITI1c after IN&OUT PCR from *a* showing minimal insertions at the left and minimal deletions at the right junction. c, Beta-2 Microglobulin surface expression after knock-out using three different gRNA constructs (*n* = 2 independent donors). d-f, GD2-CAR knock-in into *TRAC* and *B2M. *Quadrant flow plots (d), GD2-CAR frequency (e) and yield (f) across three independent donors assessed on day 10. Error bars indicate SD. **Supplementary Fig. 3. **Optimization of surface-marker based enrichment after CRISPR knock-in. a+b, Comparison of post enrichment purity of GD2-CAR-tEGFR knock-in T cells using column free (Stemcell) and column based (Miltenyi) magnetic selection. CAR+ T cells were enriched on day 14. a, Representative quadrant flow plots from pre and post enrichment samples. b, Post enrichment Purity. c, Comparison of mid culture (day 9) and harvest (day 14) enrichment. Indicated are the pre and post enrichment purity of GD2-CAR-tEGFR knock-in T cells assessed on day 14. d, Post enrichment Viability (determined on day 14) for mid culture and harvest enriched GD2-CAR-tEGFR knock-in T cells. e, GD2-CAR-T cell yield normalized to electroporated number of T cells (day 14). f, Total T cell, and CAR+ T cell counts (day 14). All experiments were conducted with *n* = 2 independent donors and d-f were analyzed using technical duplicates. Error bars indicate SD. **Supplementary Fig. 4. **Comparison of clinically established enrichment platforms reveals increased cell yields when DHFR-FS is knocked-in along with an GD2-CAR. a, GD2-CAR frequencies pre and post enrichment as determined via flow cytometry on day 14. GD2-CAR-tNGFR and GD2-CAR-tEGFR knock-in cells were enriched on day 9 using column based magnetic selection. b, Viability on day 14. c, total GD2-CAR-T cells counts on day 14 post enrichment. All experiments were conducted with *n* = 2 independent donors. **Supplementary Fig. 5. **HITI/CEMENT enables CRISPR knock-in and enrichment of functional GPC2 knock-in CAR-T cells. a, Representative qudrant flow plot showing GPC2 CAR expression on day 10 in *TRAC* knock-out cells without MTX enrichment (left) or after enrichment in 50nM MTX from day 3-7 (right). b, GPC2 CAR expression on day 10 after HITI mediated knock-in without (-MTX) or with enrichment (+MTX). c, GPC2 CAR yields on day 14 relative to electroporated cells (*n* = 3 independent donors). **Supplementary Fig. 6.** Feasibility of HITI based CRISPR knock-in for clinical manufacturing. a, Enriched CD3+ T cell counts from adult and pediatric patient leukapheresis treated with viral CAR-T cells across different trials and manufactured at two sites. Dashed line indicates number of cells activated per condition (+/-MTX) in CRISPR knock-in scale up experiments. b, Day 2 T cells counts (post activation induced contraction) normalized to number of activated T cells for viral GD2-CAR-T cell manufacturing and our proposed CRISPR knock-in CAR-T cell manufacturing process. c, Overview of T cell numbers, reagent volumes and concentrations used for large scale electroporations. d+e, Post manufacturing viability (d) and normalized cell counts (e) of CRISPR knock-in CAR-T cells from non-enriched and MTX enriched donors (*n* = 2 independent donors, counts from technical duplicates) either cultivated in media supplemented with IL-7/IL-15 or without cytokines. Statistical analysis performed with repeated measures ANOVA. Error bars indicate SD. **Supplementary Fig. 7.** Gating strategies. Gating strategies for CRISPR GD2-CAR knock-in T cells into *TRAC *(a), for viability of edited vs. non-edited T cells after knock-in GD2-CAR-DHFR-FS and MTX selection on day 7 (b), for phenotype, memory and exhaustion marker characterization (c) and activation marker and intracellular cytokines post co-culture with GD2 expressing cell lines (d). **Supplementary Fig. 8.** CAR and tumor antigen expression. CAR expression levels of viral transduced GD2-CAR and CRISPR knock-in GD2-CAR-DHFR-FS as indicated by histograms (a), median fluorescence intensity (MFI, b) and coefficient of variation (c) (*n* = 3 independent donors from Fig. 3). Differences were evaluated for statistical significance by paired, two-tailed *t* tests. d, GD2 antigen levels on tumor cell lines described in Fig. 4. Molecule count/cell determined via Quantibrite beads (*n* = 3 independent experiments). e+f, IL2 (e) and IFNg (f) secretion 24 hours after co-culture with respective GD2 antigen expressing tumor cell lines assessed via ELISA (*n* = 2 independent donors, each donor was analyzed using technical triplicates). g, Intracellular cytokine (TNF-a, IL-2, IFN-g) and activation marker (CD107a, CD69) expression after 6 hours of co-culture with respective GD2 expressing tumor cell lines. Shown here is the marker positive cell frequency gated on CD4+ CAR+ T cells (*n* = 2 independent donors, each donor was analyzed using technical triplicates). Error bars indicate SD. **Supplementary Fig. 9.** rhAmpSeq sequencing quality control metrics and CRISPAltRations results for Donors 1 and 2. a, Genomic distribution of predicted off–target sites indicating no cutting within an Exon. b, Percentage of total reads that passed QC, were merged and mapped exceeded 95%, and primer dimers were found in <1% of samples. c, Total read counts per sample and d, per target indicating sufficient coverage. For OT 30 and 31 (both located on choromosome Y) results from the female Donor 1 were excluded from the analysis. e-g, CRISPAltRations results as shown in Fig. 6c-e for Donors 1 and 2 of the large-scale experiments. Error bars indicate SD. **Supplementary Fig. 10.** TLA confirms on-target insertion. a-d, Genome-wide insertion site analysis indicates targeted insertion into *TRAC* locus at chromosome 14 across non-enriched (a+c) and enriched (b+d) samples from two additional, independent donors.

**Additional file 2. **

## Data Availability

The main data supporting the findings of this study are available within the article and its Supplementary information. All raw data generated during the study are available from the corresponding authors upon reasonable request.

## Abbreviations

HITI: Homology-independent targeted insertion
CAR: Chimeric antigen receptor
SOC: Standard-of-care
dsDNA: Double-stranded DNA
ssDNA: Single-stranded DNA
HDR: Homology directed repair
*TRAC*: T cell receptor alpha constant
RNP: Ribonucleoprotein
AAV: Adeno-associated virus
NHEJ: Non-homologous end joining
DHFR: Dihydrofolate Reductase
MTX: Methotrexate
tEGFR: Truncated Epidermal Growth Factor Receptor
tNGFR: Truncated Nerve Growth Factor Receptor
ddPCR: Digital droplet PCR
ELISA: Enzyme-linked immunosorbent assay
EFS: EF-1a short sequence
*B2M*: Beta 2 Microglobulin
TLA: Targeted Locus Amplification
GPC-2: Glypican-2
neo TCR: Neoantigen-secific T-cell receptor
tCTS: Truncated Cas9 target sequence
CEMENT: CRISPR knock-in EnrichMENT
