# Supplementary material for: Homology-independent targeted insertion (HITI) enables guided CAR knock-in and efficient clinical scale CAR-T cell manufacturing
Source: Mol Cancer. 2023 Jun 26;22:100. doi: 10.1186/s12943-023-01799-7 (PMC10291796; doi:10.1186/s12943-023-01799-7)
Supplement: Supplementary file 1 — Additional file 1: Supplementary Fig. 1. Concentration optimization of CRISPR knock-in nanoplasmid constructs. a, Cell viability after electroporation with indicated amounts of nanoplasmid per 1x10^6^ cells. b, GD2-CAR expression after electroporation with indicated amount of nanoplasmid (n = 2 independent donors). Supplementary Fig. 2. CRISPR knock-in using HITI1c integrates electroporated nanoplasmid DNA and can be applied in a versatile manner. a, IN&OUT PCR of genomic DNA extracted from Mock, HITI1c and HDR2c samples using primers targeting the endogenous TRAC sequence outside homology arms of HDR2c and sequences within knock-in templates. b, Mapped Sanger Sequencing results from HITI1c after IN&OUT PCR from a showing minimal insertions at the left and minimal deletions at the right junction. c, Beta-2 Microglobulin surface expression after knock-out using three different gRNA constructs (n = 2 independent donors). d-f, GD2-CAR knock-in into TRAC and B2M. Quadrant flow plots (d), GD2-CAR frequency (e) and yield (f) across three independent donors assessed on day 10. Error bars indicate SD. Supplementary Fig. 3. Optimization of surface-marker based enrichment after CRISPR knock-in. a+b, Comparison of post enrichment purity of GD2-CAR-tEGFR knock-in T cells using column free (Stemcell) and column based (Miltenyi) magnetic selection. CAR+ T cells were enriched on day 14. a, Representative quadrant flow plots from pre and post enrichment samples. b, Post enrichment Purity. c, Comparison of mid culture (day 9) and harvest (day 14) enrichment. Indicated are the pre and post enrichment purity of GD2-CAR-tEGFR knock-in T cells assessed on day 14. d, Post enrichment Viability (determined on day 14) for mid culture and harvest enriched GD2-CAR-tEGFR knock-in T cells. e, GD2-CAR-T cell yield normalized to electroporated number of T cells (day 14). f, Total T cell, and CAR+ T cell counts (day 14). All experiments were conducted with n = 2 independent donors and d-f were analy [file 12943_2023_1799_MOESM1_ESM.docx]

**Supplementary Fig. 1: Concentration optimization of CRISPR knock-in nanoplasmid constructs**

a, Cell viability after electroporation with indicated amounts of nanoplasmid per 1x10^6^ cells. b, GD2-CAR expression after electroporation with indicated amount of nanoplasmid (*n* = 2 independent donors).

**Supplementary Fig. 2: CRISPR knock-in using HITI1c integrates electroporated nanoplasmid DNA and can be applied in a versatile manner**

a, IN&OUT PCR of genomic DNA extracted from Mock, HITI1c and HDR2c samples using primers targeting the endogenous *TRAC* sequence outside homology arms of HDR2c and sequences within knock-in templates. b, Mapped Sanger Sequencing results from HITI1c after IN&OUT PCR from *a* showing minimal insertions at the left and minimal deletions at the right junction. c, Beta-2 Microglobulin surface expression after knock-out using three different gRNA constructs (*n* = 2 independent donors). d-f, GD2-CAR knock-in into *TRAC* and *B2M.* Quadrant flow plots (d), GD2-CAR frequency (e) and yield (f) across three independent donors assessed on day 10. Error bars indicate SD.

**Supplementary Fig. 3: Optimization of surface-marker based enrichment after CRISPR knock-in.**

a+b, Comparison of post enrichment purity of GD2-CAR-tEGFR knock-in T cells using column free (Stemcell) and column based (Miltenyi) magnetic selection. CAR+ T cells were enriched on day 14. a, Representative quadrant flow plots from pre and post enrichment samples. b, Post enrichment Purity. c, Comparison of mid culture (day 9) and harvest (day 14) enrichment. Indicated are the pre and post enrichment purity of GD2-CAR-tEGFR knock-in T cells assessed on day 14. d, Post enrichment Viability (determined on day 14) for mid culture and harvest enriched GD2-CAR-tEGFR knock-in T cells. e, GD2-CAR-T cell yield normalized to electroporated number of T cells (day 14). f, Total T cell, and CAR+ T cell counts (day 14). All experiments were conducted with *n* = 2 independent donors and d-f were analyzed using technical duplicates. Error bars indicate SD.

**Supplementary Fig. 4: Comparison of clinically established enrichment platforms reveals increased cell yields when DHFR-FS is knocked-in along with an GD2-CAR**

a, GD2-CAR frequencies pre and post enrichment as determined via flow cytometry on day 14. GD2-CAR-tNGFR and GD2-CAR-tEGFR knock-in cells were enriched on day 9 using column based magnetic selection. b, Viability on day 14. c, total GD2-CAR-T cells counts on day 14 post enrichment. All experiments were conducted with *n* = 2 independent donors.

**Supplementary Fig. 5: HITI/CEMENT enables CRISPR knock-in and enrichment of functional GPC2 knock-in CAR-T cells**

a, Representative qudrant flow plot showing GPC2 CAR expression on day 10 in *TRAC* knock-out cells without MTX enrichment (left) or after enrichment in 50nM MTX from day 3-7 (right). b, GPC2 CAR expression on day 10 after HITI mediated knock-in without (-MTX) or with enrichment (+MTX). c, GPC2 CAR yields on day 14 relative to electroporated cells (*n* = 3 independent donors).

**Supplementary Fig. 6: Feasibility of HITI based CRISPR knock-in for clinical manufacturing**

a, Enriched CD3+ T cell counts from adult and pediatric patient leukapheresis treated with viral CAR-T cells across different trials and manufactured at two sites. Dashed line indicates number of cells activated per condition (+/-MTX) in CRISPR knock-in scale up experiments. b, Day 2 T cells counts (post activation induced contraction) normalized to number of activated T cells for viral GD2-CAR-T cell manufacturing and our proposed CRISPR knock-in CAR-T cell manufacturing process. c, Overview of T cell numbers, reagent volumes and concentrations used for large scale electroporations. d+e, Post manufacturing viability (d) and normalized cell counts (e) of CRISPR knock-in CAR-T cells from non-enriched and MTX enriched donors (*n* = 2 independent donors, counts from technical duplicates) either cultivated in media supplemented with IL-7/IL-15 or without cytokines. Statistical analysis performed with repeated measures ANOVA. Error bars indicate SD.

**Supplementary Fig. 7: Gating strategies**

Gating strategies for CRISPR GD2-CAR knock-in T cells into *TRAC* (a), for viability of edited vs. non-edited T cells after knock-in GD2-CAR-DHFR-FS and MTX selection on day 7 (b), for phenotype, memory and exhaustion marker characterization (c) and activation marker and intracellular cytokines post co-culture with GD2 expressing cell lines (d).

**Supplementary Fig. 8: CAR and tumor antigen expression**

CAR expression levels of viral transduced GD2-CAR and CRISPR knock-in GD2-CAR-DHFR-FS as indicated by histograms (a), median fluorescence intensity (MFI, b) and coefficient of variation (c) (*n* = 3 independent donors from Fig. 3). Differences were evaluated for statistical significance by paired, two-tailed *t* tests. d, GD2 antigen levels on tumor cell lines described in Fig. 4. Molecule count/cell determined via Quantibrite beads (*n* = 3 independent experiments). e+f, IL2 (e) and IFNg (f) secretion 24 hours after co-culture with respective GD2 antigen expressing tumor cell lines assessed via ELISA (*n* = 2 independent donors, each donor was analyzed using technical triplicates). g, Intracellular cytokine (TNF-a, IL-2, IFN-g) and activation marker (CD107a, CD69) expression after 6 hours of co-culture with respective GD2 expressing tumor cell lines. Shown here is the marker positive cell frequency gated on CD4+ CAR+ T cells (*n* = 2 independent donors, each donor was analyzed using technical triplicates). Error bars indicate SD.

**Supplementary Fig. 9: rhAmpSeq sequencing quality control metrics and CRISPAltRations results for Donors 1 and 2**

a, Genomic distribution of predicted off–target sites indicating no cutting within an Exon. b, Percentage of total reads that passed QC, were merged and mapped exceeded 95%, and primer dimers were found in <1% of samples. c, Total read counts per sample and d, per target indicating sufficient coverage. For OT 30 and 31 (both located on choromosome Y) results from the female Donor 1 were excluded from the analysis. e-g, CRISPAltRations results as shown in Figure 6 c-e for Donors 1 and 2 of the large-scale experiments. Error bars indicate SD.

**Supplementary Fig. 10: TLA confirms on-target insertion**

a-d, Genome-wide insertion site analysis indicates targeted insertion into *TRAC* locus at chromosome 14 across non-enriched (a+c) and enriched (b+d) samples from two additional, independent donors.
